# *In silico* characterisation, homology modelling and structure-based functional annotation of blunt snout bream (*Megalobrama amblycephala*) Hsp70 and Hsc70 proteins

**DOI:** 10.1186/s40781-015-0077-x

**Published:** 2015-12-15

**Authors:** Ngoc Tuan Tran, Ivan Jakovlić, Wei-Min Wang

**Affiliations:** College of Fisheries, Key Lab of Agricultural Animal Genetics, Breeding and Reproduction of Ministry of Education/Key Lab of Freshwater Animal Breeding, Ministry of Agriculture, Huazhong Agricultural University, Wuhan, Hubei 430070 China; Collaborative Innovation Center for Efficient and Health Production of Fisheries in Hunan Province, Changde, 41500 China; Center for Fish Biology and Fishery Biotechnology, Institute of Hydrobiology, Chinese Academy of Sciences, Wuhan, Hubei, 430072 China

**Keywords:** Hsp70, Hsc70, Physicochemical characteristics, Homology modelling, Structural similarity, Functional annotation

## Abstract

**Background:**

Heat shock proteins play an important role in protection from stress stimuli and metabolic insults in almost all organisms.

**Methods:**

In this study, computational tools were used to deeply analyse the physicochemical characteristics and, using homology modelling, reliably predict the tertiary structure of the blunt snout bream (*Ma*-) Hsp70 and Hsc70 proteins. Derived three-dimensional models were then used to predict the function of the proteins.

**Results:**

Previously published predictions regarding the protein length, molecular weight, theoretical isoelectric point and total number of positive and negative residues were corroborated. Among the new findings are: the extinction coefficient (33725/33350 and 35090/34840 - *Ma*-Hsp70/ *Ma*-Hsc70, respectively), instability index (33.68/35.56 – both stable), aliphatic index (83.44/80.23 – both very stable), half-life estimates (both relatively stable), grand average of hydropathicity (−0.431/-0.473 – both hydrophilic) and amino acid composition (alanine-lysine-glycine/glycine-lysine-aspartic acid were the most abundant, no disulphide bonds, the N-terminal of both proteins was methionine). Homology modelling was performed by SWISS-MODEL program and the proposed model was evaluated as highly reliable based on PROCHECK’s Ramachandran plot, ERRAT, PROVE, Verify 3D, ProQ and ProSA analyses.

**Conclusions:**

The research revealed a high structural similarity to Hsp70 and Hsc70 proteins from several taxonomically distant animal species, corroborating a remarkably high level of evolutionary conservation among the members of this protein family. Functional annotation based on structural similarity provides a reliable additional indirect evidence for a high level of functional conservation of these two genes/proteins in blunt snout bream, but it is not sensitive enough to functionally distinguish the two isoforms.

## Background

Heat shock (or stress) proteins (HSPs) are a family of highly conserved cellular proteins that play an important role in protection from stress stimuli and metabolic insults in almost all organisms [1–4]. They include three major families: Hsp90 (85–90 kDa), Hsp70 (68–73 kDa) and low molecular-weight Hsps (16–47 kDa) [3]. The Hsp70 family is encoded by two different genes: a constitutive, “housekeeping” *heat shock cognate* (*hsc*) *70* gene, which is predominantly associated with physiological processes, and stress-inducible *hsp70*. Hsc70 protein plays a key role as molecular chaperone in a wide range of cellular processes, such as protein assembly, folding, transport through membrane channels, translocation and denaturation [4–7]. Hsp70 protein is mainly responsible for the maintenance of cellular homeostasis during the stress response, thus protecting cells from the damage caused by environmental stress agents, such as heat shock, chemical exposure and UV or γ-irradiation [4, 8, 9]. Hsp70 is also a potential activator of the innate immune system mechanisms [10–12]. Hsp70 is considered to be by far the most evolutionary conserved protein, found in all organisms from archaebacteria and plants to humans [13]. Both proteins have a modular structure consisting of a highly conserved N-terminal ATPase domain, an adjacent well-conserved substrate-binding domain (SBD) that contains a hydrophobic pocket with a lid-like structure over it, and a conserved but more variable C-terminal domain, which plays an important role in Hsp70 functions required for cell growth. In the ATP-bound state, the substrate-binding pocket is open and rapidly exchanges substrate. ATP hydrolysis induces closing of the lid over the pocket, which stabilises substrate binding. Return to the ATP-bound state restores the open conformation, facilitating substrate release [14, 15].

In aquaculture, fish are often exposed to stressful situations, such as sudden temperature changes, high stocking density, trauma, hypoxia, as well as viral and bacterial infections, which often results in high fish mortality. Both genes have been identified and their expression characterised in many fish species, e.g., rainbow trout (*Oncorhynchus mykiss*), zebrafish (*Danio rerio*), Korean rockfish (*Sebastes schlegeli*), Nile tilapia (*Oreochromis niloticus*), mandarin fish (*Siniperca chuatsi*) [16–20] and both are known to have a crucial role in response to heat shock, hypoxia, crowding stress and bacterial pathogens in fish [3, 4, 7, 19]. However, according to our best knowledge, a three-dimensional (3-D) model of any fish Hsp70 family protein has not been published so far.

Blunt snout bream (*Megalobrama amblycephala* Yih, 1955), native to the middle portions of the Yangtze River basin, is becoming an increasingly important freshwater aquaculture species in China. Due to its successful artificial propagation and high economic value, the total output of the blunt snout bream aquaculture industry reached 652 215 tons in 2010 [21–23]. Previously, Ming, Xie [7] used bioinformatics tools to analyse some physicochemical characteristics of the two *Ma*-Hsp70 family proteins, such as molecular weight, isoelectric point, solubility (as hydrophilic property) and richness in B cells antigenic sites. Their results indicated that *Ma*-Hsp70 shares more than 85 % identity with its homologs in other vertebrates, has no signal peptide or transmembrane region, contains many protein kinase C phosphorylation sites, N-myristoylation sites, casein kinase II phosphorylation sites and N-glycosylation sites, while the predominant elements of the secondary structure are α-helix and random coil. However, as the previous study left many questions regarding the physicochemical and structural properties (particularly regarding the tertiary structure) open, this study, as a successive work, aims to fill this gap. Several different computational tools and available web servers were used to deeply analyse the physicochemical characteristics and, using homology modelling, reliably predict the tertiary structure of the blunt snout bream Hsp70 and Hsc70 proteins. Additionally, rapidly increasing number of known gene sequences in many organisms has prompted the need for new procedures and techniques for the high-throughput functional annotation of genes. While most of those traditionally used remain rather costly and work-intensive, with rapidly growing number of protein structures deposited in the Protein Data Bank (PDB), computational structural genomics is becoming an increasingly promising tool for fast and cheap insight into protein structures, functions and interactions [24–27]. As Ming, Xie [7] analysed the expression of *Ma*-*hsp70* and *Ma*-*hsc70* genes in order to gain insight into their functions, this study further aims to provide a supplementary evidence for conserved function of these two genes by in-deep structure analysis and functional annotation of their polypeptide products on the basis of the similarity of their tertiary structures to the available templates from other organisms. As structure-based functional annotation has seldom been used in study of fish proteins, the aim of this study is also to test the applicability of this approach for functional annotation of fish gene sequences.

## Methods

### Physicochemical characterisation

Amino acid sequences of the blunt snout bream Hsp70 (Accession number: ACG63706.2) and Hsc70 (Accession number: GQ214528.1) [7] were obtained from the NCBI protein database (http://www.ncbi.nlm.nih.gov/) in FASTA format as the target template and used for further analyses. Physicochemical properties of the proteins, including molecular weight, amino acid composition, theoretical isoelectric point (pI), the total number of positive and negative residues, extinction coefficient (EC), instability index (II), aliphatic index (AI) and grand average of hydropathicity (GRAVY) were analysed using Expasy’s ProtParam prediction server [28]. SOSUI server [29] was used to determine whether it is a soluble or a transmembrane protein, while CYS_REC (http://linux1.softberry.com) was used to predict the presence of cysteine residues and their bonding patterns.

### Comparative homology modelling

Homology modelling of the proteins was performed by the SWISS-MODEL server [30, 31], which aligns an input target with pre-existing templates to generate a series of predicted models. The most suitable template to build the 3-D model was selected on the basis of sequence identity [32]. Multiple amino acid sequence alignment was performed with ClustalW2 (http://www.ebi.ac.uk/Tools/msa/clustalw2). Stereochemical quality and accuracy of the predicted models were analysed using PROCHECK’s Ramachandran plot analysis, ERRAT, PROVE, Verify3D (all four available from the SAVES server at http://nihserver.mbi.ucla.edu), ProQ [33] and ProSA [34, 35]. Structural analysis was performed and model figures generated by Swiss PDB Viewer [36].

### Structural similarity and functional annotation

COFACTOR web server was used to perform the global structure match using TM-align algorithm and render the TM-score was calculated to assess the global structural similarity: values range from 0 to 1, where TM-score = 1 indicates the perfect match between two structures. Scores below 0.17 correspond to randomly chosen unrelated proteins, whereas a score higher than 0.5 implies generally the same fold [37]. Annotations on ligand-binding sites, gene ontology and enzyme commission were performed by the I-TASSER suite, which structurally matches the 3-D model of *Ma*-Hsp70 and *Ma*-Hsc70 to the known templates in protein function databases [38–40].

## Results and discussion

### Physicochemical characterisation

In this study, several different computational tools and available web servers were used to deeply analyse the physicochemical characteristics and to reliably predict the tertiary structure of the blunt snout bream Hsp70 and Hsc70 proteins (using homology modelling). This research corroborated the previous predictions regarding the *Ma*-Hsp70 and *Ma*-Hsc70 protein length (643 and 649 amino acids), molecular weight (70517.7 and 71240.3 Da), theoretical isoelectric point (pI = 5.36 and 5.31) and the total number of negatively and positively charged residues (94 and 81 and 96 and 82, respectively) [7]. Among the new findings, the computed pI value indicated that the proteins are acidic (pI < 7) in character, implying that they can be purified on a polyacrylamide gel by isoelectric focusing. The calculated extinction coefficient (EC), which is in direct correlation with the cysteine, tryptophan and tyrosine content, of *Ma*-Hsp70 and *Ma*-Hsc70 proteins at 280 nm was 33725/33350 (assuming all pairs of cysteine residues form cysteines) and 35090/34840 (assuming all cysteine residues are reduced) M^−1^cm^−1^, respectively. The instability index (II) value was 33.68 (*Ma*-Hsp70) and 35.56 (*Ma*-Hsc70), implying that both proteins are stable (II < 40) [41]. Similarly, the aliphatic index (AI) of both proteins had very high value of 83.44 (*Ma*-Hsp70) and 80.23 (*Ma*-Hsc70), indicating stability over a wide temperature range [42]. The N-terminal of both proteins was methionine. Estimated half-life values were also the same for both proteins: 30 h in mammalian reticulocytes (in vitro), >20 h in yeast (in vivo) and >10 h in *Escherichia coli* (in vivo). The grand average hydropathicity (GRAVY) of *Ma*-Hsp70 and *Ma*-Hsc70 was −0.431 and −0.473, respectively, corroborating that proteins are hydrophilic and highly soluble in water. Amino acid composition analysis revealed high amounts of alanine (8.4 %), lysine (8.2 %) and glycine (8.1 %) in *Ma*-Hsp70, whereas glycine (8.5 %), lysine (8.2 %) and aspartic acid (7.6 %) were the most abundant in *Ma*-Hsc70 (Table 1).

**Table 1:** Amino acid composition of *Ma*-Hsp70 and *Ma*-Hsc70

Amino acid	*Ma*-Hsp70		*Ma*-Hsc70		Amino acid	*Ma*-Hsp70		*Ma*-Hsc70	
	N	%	N	%		N	%	N	%
Alanine	54	8.4	47	7.2	Lysine	53	8.2	53	8.2
Arginine	28	4.4	29	45	Methionine	14	2.2	15	2.3
Asparagine	36	5.6	34	5.2	Phenylalanine	22	3.4	23	3.5
Aspartic acid	47	7.3	49	7.6	Proline	20	3.1	25	3.9
Cysteine	6	0.9	4	0.6	Serine	36	5.6	37	5.7
Glutamine	28	4.4	25	3.9	Threonine	41	6.4	48	7.4
Glutamic acid	47	7.3	47	7.2	Tryptophan	2	0.3	2	0.3
Glycine	52	8.1	55	8.5	Tyrosine	15	2.3	16	2.5
Histidine	7	1.1	7	1.1	Valine	44	6.8	45	6.9
Isoleucine	45	7.0	46	7.1	Pyrrolysine	0	0.0	0	0.0
Leucine	46	7.2	42	6.5	Selenocysteine	0	0.0	0	0.0

N represents the total number and % the numeric percentage of each amino acid

Though six and four cysteine residues were found in *Ma*-Hsp70 and *Ma*-Hsc70 sequences, respectively, no evidence was found for the existence of disulphide bonds, which are essential for the folding of proteins and responsible for stabilisation of protein structure [43] (Table 2).

**Table 2:** Cysteine occurrence pattern and probability of cysteine residue pairing in *Ma*-Hsp70 and *Ma*-Hsc70 proteins

Protein	Position	Status	Score
*Ma*-Hsp70	Cys19	no SS-bond	−58.8
	Cys269	no SS-bond	−34.4
	Cys308	no SS-bond	−24.9
	Cys576	no SS-bond	−29.6
	Cys605	no SS-bond	−27.5
	Cys622	probably no SS-bond	−8.00
*Ma*-Hsc70	Cys17	no SS-bond	−55.6
	Cys267	no SS-bond	−37.8
	Cys574	no SS-bond	−32.2
	Cys603	no SS-bond	−23.6

### Comparative homology modelling

**Fig. 1:**
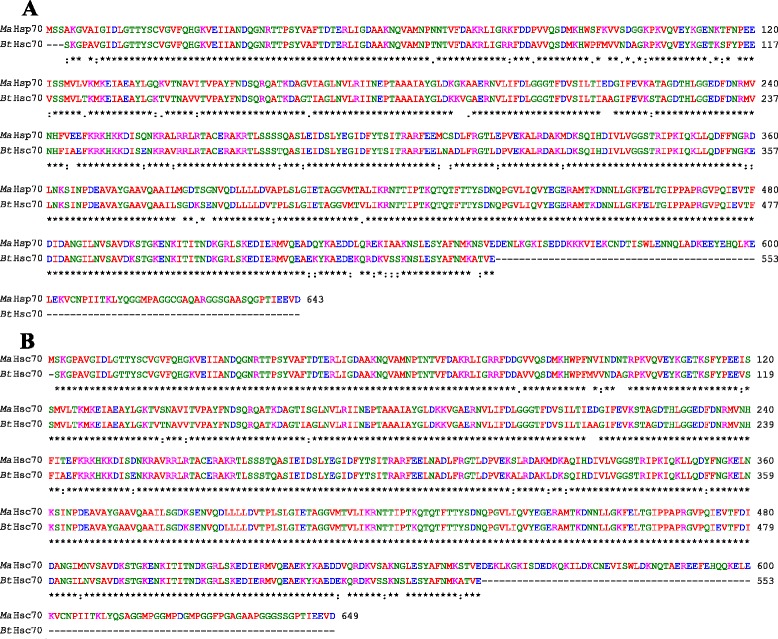
Alignment of the deduced *Ma*-Hsp70 **a** and *Ma*-Hsc70 **b** with bovine Hsc70 (PDB ID: 4 fl9.1.A) amino acid sequence. Amino acid positions are numbered on the right, conserved substitutions are indicated by (:), semi-conserved by (.) and deletions by (−)

**Fig. 2:**
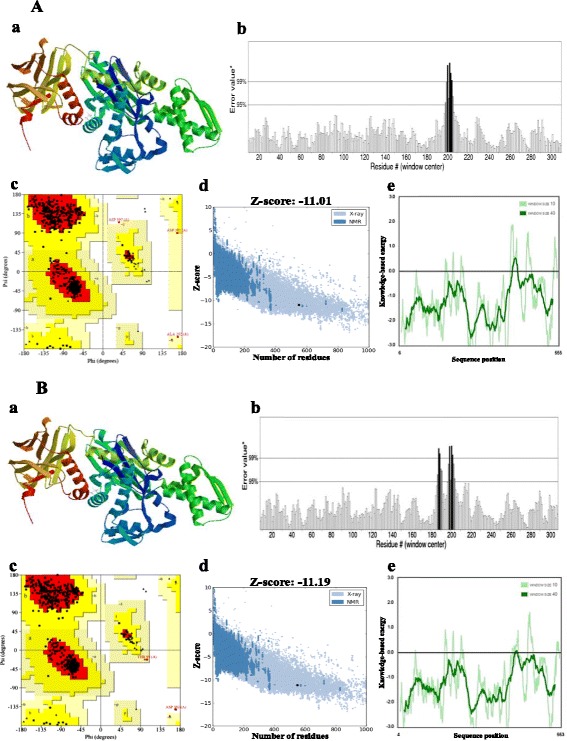
*Ma*-Hsp70 **a** and *Ma*-Hsc70 **b** protein tertiary structure model and validation results: **a** 3-D homology model rendered by the SWISS-MODEL program. **b** Ramachandran plot analysis, indicating residues in the favoured regions (red), allowed regions (yellow), generously allowed regions (light yellow) and disallowed regions (white). **c** Overall quality of the model evaluated by the ERRAT program. On the error axis, two lines (95 and 99 %) indicate the confidence with which it is possible to reject regions that exceed that error value. Regions of the structure highlighted in grey and black can be rejected at 95 % and 99 % confidence level, respectively. **d** Z-score (highlighted as a black dot) is displayed in a plot that contains the Z-scores of all experimentally determined protein chains currently available in the Protein Data Bank. Groups of structures from different sources (X-ray and NMR) are distinguished by different colours (light- and dark-blue, respectively). **e** Plot of single residue energies, where window sizes of 40 and 10 residues are distinguished by dark- and light-green lines, respectively. Positive values indicate problematic or erroneous parts of the structure

Bovine Hsc70 (PDB ID: 4 fl9.1.A) at 1.9 Å resolution was chosen as the best available template to build a 3-D model for both *Ma*-Hsp70 (90.24 % sequence identity) and *Ma*-Hsc70 (95.85 % seq. identity) proteins using homology modelling. Template-target sequence alignments and 3-D structure of the predicted models are shown in Figs. 1 and 2, respectively. Verification of the results, using different tools, invariably indicated a good quality of the proposed models (Table 3). So the Ramachandran plot analysis, where a good model would be expected to have over 90 % of residues in the most favoured regions, suggested a good quality of both homology models (*Ma*-Hsp70 - 92.7 % and *Ma*-Hsc70 - 92.4 %). The overall G-factor of the two models, where a value > −0.5 indicates a good model [44], was 0.16. Verify3D analysis of the models revealed that 92.55 % (*Ma*-Hsp70) and 92.36 % (*Ma*-Hsc70) of the residues had an average 3D-1D score ≥0.2, while 98.36 % and 99.82 % were >0, respectively. As the cut-off score was ≥0, this implies the predicted models are valid [45]. Overall ERRAT quality factor value, expressed as the percentage of the protein for which the calculated value falls below the 95 % rejection limit, was 95.547 % and 92.963 %, respectively (Fig. 2). Good high resolution structures generally produce values around 95 % or higher [46]. LGscore and MaxSub index indicated “very good” (value >5.0) and “correct” quality (value >0.1) [33] for *Ma*-Hsp70 and *Ma*-Hsc70 protein models, respectively (Table 3). Z-scores for *Ma*-Hsp70 (−11.01) and *Ma*-Hsc70 (−11.19) models were within the range of scores typically found for the native proteins of similar size, while the plot of residue energies, where positive values correspond to problematic or erroneous parts of the input structure, revealed that most of the calculated values were negative [35] (Fig. 2). All of these validation tools strongly suggested that both proposed 3-D models could be accepted as reliable with high confidence.

**Table 3:** Assessment of the predicted three-dimensional structures of *Ma*-Hsp70 and *Ma*-Hsc70 proteins

Validation Index	*Ma*-Hsp70	*Ma*-Hsc70
Ramachandran plot		
Residues in most favoured regions	92.7	92.4
Residues in additional allowed regions	6.7	7.1
Residues in generously allowed regions	0.4	0.2
Residues in disallowed regions	0.2	0.2
Overall G-factor	0.16	0.16
ProQ		
Lgscore	5.417	5.467
MaxSub	0.448	0.427
ProSA Z-Score	−11.01	−11.19
ERRAT	95.547	92.63

### Structure similarity analysis

**Fig. 3:**
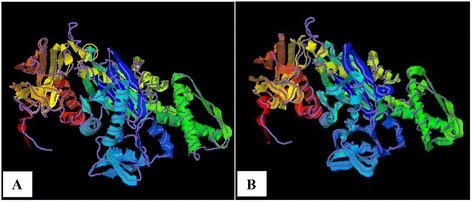
*Bos taurus* Hsc70 (PDB ID: 1yuw-A) structural analog (backbone trace) superimposed upon the *Ma*-Hsp70 **a** and *Ma*-Hsc70 **b** proteins (shown in cartoon), rendered by COFACTOR server

As TM-scores >0.5 indicate that two proteins generally have the same fold, the results (TM > 0.66) implied a very high level of structural conservation between *Ma*-Hsp70 and related rat, mouse, human and yeast proteins (Table 4). TM-score value of 0.986 between both *Ma*-Hsp70 and *Ma*-Hsc70 and *Bos taurus* Hsc70 3-D protein models indicates that they are structurally almost identical (Fig. 3). Somewhat surprisingly, TM-scores were identical for both protein models, while RMSD^a^ IDEN^a^ and Cov. values were different between the two models. This could be explained by the fact that their tertiary structure is more similar than their primary and secondary structure, as well as by the absence of fish Hsp/Hsc70 templates in the PDB. The fact that the same PDB protein models (three Hsc and two Hsp) were indicated as the best available templates for both tested models reflects a very high level of conservation among the two proteins and other vertebrate Hsp70 family proteins (*Ma*-Hsp70 - 86 % identity and *Ma*-Hsc70 - 93 %), as well as between *Ma*-Hsp70 and *Ma*-Hsc70 (86.5 % identity) [7]. High structural similarity to Hsp70 protein family members from a wide range of taxonomically distant organisms additionally corroborated a remarkably high level of evolutionary conservation among the members of this protein family and provided an indirect evidence for a high level of functional conservation as well.

**Table 4:** Top five identified structural analogs in the Protein Data Bank (PDB) library

	PDB ID	Protein	Species	TM-score	RMSD^a^	IDEN^a^	Cov.
*Ma*-Hsp70	1yuwA	Hsc70	*Bos taurus*	0.986	0.95	0.896	0.996
	4j8fA	Hsc70	*Rattus norvegicus*	0.688	1.78	0.676	0.711
	3cqxB	Hsc70	*Mus musculus*	0.678	0.90	0.902	0.685
	3iucC	Hsp70	*Homo sapiens*	0.677	0.95	0.687	0.685
	3qmlA	Hsp70	*Saccharomyces cerevisiae*	0.669	1.17	0.656	0.682
*Ma*-Hsc70	1yuwA	Hsc70	*Bos taurus*	0.986	1.24	0.958	1.000
	4j8fA	Hsc70	*Rattus norvegicus*	0.688	1.81	0.654	0.711
	3cqxB	Hsc70	*Mus musculus*	0.678	0.90	0.960	0.685
	3iucC	Hsp70	*Homo sapiens*	0.677	0.95	0.698	0.685
	3qmlA	Hsp70	*Saccharomyces cerevisiae*	0.669	1.17	0.659	0.682

Analogs were inferred by COFACTOR analysis, based on the TM-score of the structural alignment between the query structure and known structures in the PDB. RMSD^a^ is the average root mean square deviation between residues that are structurally aligned by TM-align; IDEN^a^ is the percentage sequence identity in the structurally aligned region; Cov. represents the coverage of the alignment by TM-align and is equal to the number of structurally aligned residues divided by length of the query protein

### Function prediction on the basis of structural similarity

An ATP-binding site was predicted with a very high confidence, on the basis of structure similarity with bovine Hsc70 (PDB ID = 1kax-A; C-score = 0.98) and yeast actin (1yag-A; 0.96) for *Ma*-Hsp70 and *Ma*-Hsc70, respectively. High structural identity with yeast actin [47] reflects structural and mechanistic similarities between ATP hydrolytic mechanisms in proteins with different functions. In line with its ATPase function and previous findings [48], a phosphate ion (PO4)-binding site was also predicted with a relatively significant confidence on the basis of structural similarity with the bovine Hsc70 II (PDB ID = 1hpm-A; C-score = 0.22) for *Ma*-Hsp70 and the N-terminal domain of the *Cryptosporidium parvum* Hsp70 (PDB ID = 3l4i-B; C-score = 0.23) for *Ma*-Hsc70 (Table 5).

**Table 5:** Residue-specific ligand binding probability

	PDB ID	C-score	Clust. size	Ligand	Ligand-binding site residues
*Ma*-Hsp70	1kax-A	0.98	176	ATP	14, 15, 16, 17, 203, 204, 205, 206, 232, 270, 273, 274, 277, 340, 341, 342, 344, 345, 368
	1hpm-A	0.22	33	PO4	14, 15, 73, 149, 177, 231
*Ma*-Hsc70	1yag-A	0.96	146	ATP	12, 13, 14, 15, 17, 201, 202, 203, 204, 230, 268, 271, 272, 338, 339, 340, 342, 343, 366
	3l4i-B	0.23	32	PO4	12, 13, 71, 147, 175, 229

Predicted by COACH analysis, based on the C-score of the structural alignment between the query structure and known structures in the PDB. C-score is the confidence score of the prediction, where a higher score (min-0, max-1) indicates a more reliable prediction; Clust. size is the total number of templates in a cluster; ligand is the name of a possible binding ligand

In accordance with the predicted ligands, Enzyme Commission analysis (performed by I-TASSER program) predicted, with relatively similar confidence scores, both *Ma*-Hsp70 and *Ma*-Hsc70 to be either of the isozymes hexokinase and glucokinase, both of which can transfer an inorganic phosphate group from ATP to a substrate. Somewhat confusingly, deoxyribonuclease function was also predicted for both proteins (Table 6), however, upon closer inspection of the 3cjc PDB entry, it became obvious that this prediction was the result of an error in the data retrieval from the PDB, as the 3cjc-A chain represents actin, which is an ATPase, with an adenosine-binding site, while deoxyribonuclease is the 3cjc-D chain in the entry.

**Table 6:** Enzyme Commission (EC) predictions for *Ma*-Hsp70 and *Ma*-Hsc70 proteins

	Csc^EC^	PDB ID	TM-sc	RMSD^a^	IDEN^a^	Cov	EC No.	EC Name
*Ma*-Hsp70	0.241	3cjc-A	0.421	4.08	0.137	0.493	3.1.21.1	Deoxyribonuclease I
	0.230	1qha-A	0.405	5.2	0.08	0.509	2.7.1.1	Hexokinase
	0.222	3f9m-A	0.386	4.08	0.099	0.457	2.7.1.2	Glucokinase
	0.221	3hm8-A	0.381	4.25	0.106	0.456	2.7.1.1	Hexokinase
	0.221	2e2o-A	0.362	3.8	0.103	0.417	2.7.1.1	Hexokinase
*Ma*-Hsc70	0.221	1qha-A	0.390	5.27	0.089	0.491	2.7.1.1	Hexokinase
	0.216	3hm8-A	0.378	4.12	0.111	0.447	2.7.1.1	Hexokinase
	0.215	3f9m-A	0.382	4.08	0.110	0.452	2.7.1.2	Glucokinase
	0.185	1v4t-A	0.281	4.95	0.064	0.347	2.7.1.2 2.7.1.1	Glucokinase Hexokinase
	0.168	3cjc-A	0.411	4.13	0.131	0.484	3.1.21.1	Deoxyribonuclease I

Cscore^EC^ is the confidence score for the enzyme commission number (EC No.) prediction (0–1), TM-sc is the TM-score, Cov represents the coverage of global structural alignment and is equal to the number of structurally aligned residues divided by length of the query protein. See Table 4 for other term explanations

Similarly, consensus prediction of GO terms also suggested ATP binding, interacting selectively and non-covalently with adenosine 5'-triphosphate (GO = 0005524; ontology = molecular function) as the main function for both proteins, with very high GO-scores (0.98 for Hsc70 and 0.99 for Hsp70).

All these results are in accordance with the previously described functioning mechanisms: Hsp70s in the ATP-bound state catch and release their substrates rapidly, while Hsp70s in the ADP-bound state seize them firmly. By cycling between the ATP- and ADP-bound states, Hsp70s exert their chaperone activity [14, 49]. However, the analysis was not sensitive enough to distinguish between the functions of the constitutive (Hsc) and inducible (Hsp) isoforms. This is not a major setback, as it has been suggested that the functional difference appears to lie more in regulation of the SBD-substrate interactions than in the physical properties of the two ATPase domains [15].

## Conclusions

To help better understand the functional biology of Hsp70 and Hsc70 in blunt snout bream, several computational tools were used to analyse the physicochemical properties, generate valid homology models of both proteins and predict their functions on the basis of structural similarity to other protein templates. Apart from presenting the first published homology models of Hsp70 and Hsc70 proteins in fish, this research also revealed a high structural similarity to Hsp70/Hsc70 proteins from several taxonomically distant animal species, corroborating a remarkably high level of evolutionary conservation among the members of this protein family. Functional annotation based on structure similarity provides a reliable additional indirect evidence for a high level of functional conservation of these two genes/proteins in blunt snout bream, but it is not sensitive enough to distinguish between the two isoforms. In conclusion, even though gene function assignment based on protein structure similarity is at present somewhat limited by the number of available protein structures deposited in the PDB, it has a strong potential to become a very fast, cheap and relatively reliable technique for high-throughput gene function assignment in fish.

## Abbreviations

3-D: three-dimensional
AI: aliphatic index
EC: extinction coefficient
GO: gene ontology
GRAVY: grand average of hydropathicity
Hsc: heat shock cognate
Hsp: heat shock protein
II: instability index
PDB: protein data bank
